# Multispecific resistance of sheep trichostrongylids in Austria

**DOI:** 10.1051/parasite/2021048

**Published:** 2021-06-11

**Authors:** Florian Untersweg, Viktoria Ferner, Sandra Wiedermann, Marie Göller, Marion Hörl-Rannegger, Waltraud Kaiser, Anja Joachim, Laura Rinaldi, Jürgen Krücken, Barbara Hinney

**Affiliations:** 1 Institute of Parasitology, Department of Pathobiology, Vetmeduni Vienna Veterinärplatz 1 1210 Vienna Austria; 2 Animal Health Service (TGD) Salzburg 5020 Salzburg Austria; 3 Tierärztliche Praxisgemeinschaft Passail OG 8162 Passail Austria; 4 Laboratory of Parasitology and Parasitic Diseases, Department of Veterinary Medicine and Animal Production, University of Naples Federico II 80138 Naples Italy; 5 Institute for Parasitology and Tropical Veterinary Medicine, Freie Universität Berlin 14163 Berlin Germany

**Keywords:** Nematode, Benzimidazoles, Macrocyclic lactones, Monepantel, Faecal egg count reduction test

## Abstract

Anthelmintic overuse and failure to implement methods preventing the development and spread of anthelmintic resistance (AR) have led to an alarming increase of resistant ovine trichostrongylids worldwide. The aim of the present study was to determine whether the routine anthelmintic treatment strategy was effective, to obtain insights into the frequency of AR in trichostrongylids of sheep in Austria, and to determine the presence of different trichostrongylid genera. On 30 sheep farms, the faecal egg count reduction test (FECRT) was performed with the Mini-FLOTAC technique in two consecutive studies. In study 1, only fenbendazole and moxidectin were tested, while different compounds and products were used in study 2. Overall, 33 treatment groups were formed: 11 groups were treated with benzimidazoles (fenbendazole and albendazole), 2 groups with avermectins (ivermectin, doramectin), 18 groups with moxidectin, and two groups with monepantel. Reduced efficacy was detected in 64%, 100%, 28% and 50% of these groups, respectively. The most frequently detected genus in larval cultures was *Haemonchus*, which had been barely detected in Austria previously, followed by *Trichostrongylu*s. Multispecific resistance of trichostrongylids in Austria seems to be on the rise and *H. contortus* was detected unexpectedly frequently in comparison to previous studies. There is an urgent need to develop efficient communication strategies aimed at improving the engagement of farmers and veterinarians in sustainable parasite control.

## Abstract (fr)

La surutilisation des anthelminthiques et l’échec de la mise en œuvre de méthodes empêchant le développement et la propagation de la résistance aux anthelminthiques (RA) ont conduit à une augmentation alarmante des trichostrongylidés ovins résistants dans le monde. Le but de nos études était de déterminer si la stratégie de traitement anthelminthique de routine était efficace, d’avoir un aperçu de la fréquence de la RA chez les trichostrongylidés des moutons en Autriche et de déterminer la présence de différents genres de trichostrongylidés. Dans 30 élevages ovins, le test de réduction du nombre d’œufs fécaux (FECRT) a été réalisé avec la technique Mini-FLOTAC dans deux études consécutives. Dans l’étude 1, seuls le fenbendazole et la moxidectine ont été testés, tandis que différents composés et produits ont été utilisés dans l’étude 2. Au total, trente-trois groupes de traitement ont été formés, 11 groupes ont été traités avec des benzimidazoles (fenbendazole et albendazole), 2 groupes avec des avermectines (ivermectine, doramectine), 18 groupes avec la moxidectine et deux groupes avec le monepantel. Une efficacité réduite a été détectée dans 64 %, 100 %, 28 % et 50 % de ces groupes, respectivement. Le genre le plus fréquemment détecté dans les cultures larvaires était *Haemonchus*, qui avait été rarement détecté en Autriche auparavant, suivi de *Trichostrongylus*. La résistance multispécifique des trichostrongylidés en Autriche semble augmenter et *H. contortus* a été détecté fréquemment, de manière inattendue par rapport aux études précédentes. Il est urgent de développer des stratégies de communication efficaces visant à améliorer l’engagement des éleveurs et des vétérinaires dans le contrôle durable des parasites.

## Introduction

Trichostrongyloidea in small ruminants can severely impair animal health and productivity [3]. Common signs of trichostrongylid infections are poor weight gain, weight loss, reduced wool and milk production, diarrhoea, weakness, and ill thrift [8, 25, 29]. Severe cases can result in sudden death, especially after infections with the blood feeding *Haemonchus contortus* [2]. Recently, the annual costs of helminth infection in ruminants in Europe was estimated at €1.8 billion (€29 million in Austria) [3]. The control of gastrointestinal nematode (GIN) infections can be roughly categorised into pharmaceutical and non-pharmaceutical approaches (e.g. grazing systems, use of bioactive compounds, etc.) [30]. Modern anthelmintics were initially highly efficacious, so that treatment strategies in the past decades often relied heavily on the use of drugs in suppressive treatment approaches [34, 38]. However, these strategies resulted in selection for anthelmintic resistance (AR) [38]. The use of ineffective anthelmintics in the EU has been estimated to contribute to the cost of GIN infections to an extent of €38 million annually (€0.6 million in Austria) [3]. In order to slow down the development of AR, more sustainable treatment strategies have been designed [30]. A key pillar of sustainable treatment approaches is the regular monitoring of anthelmintic efficacy [17, 30]. Among the tests to check for AR, the faecal egg count reduction test (FECRT) is the method most broadly applied. It has the advantage of being applicable to all anthelmintic drugs available, but has the disadvantage of low sensitivity [4, 5, 20]. Furthermore, there are different views on how to standardise this test. The latest guideline of the World Association for the Advancement of Parasitology (WAAVP) for this method was published in 2006, and a new guideline is to be published soon [17]. *In vitro* methods have been developed but are less widely employed. A major challenge is the standardisation of these techniques, especially for mixed species samples from the field [5, 9]. In addition, molecular techniques are available for the detection of BZ resistance alleles [5, 23]. A recent meta-analysis of AR in Europe demonstrated that AR is widespread, but that there are also clear data gaps [27]. For research on small ruminants in Austria, more reliable estimates of the prevalence of AR are considered to be beneficial [27]. The analysis also revealed that comparability between studies is difficult due to non-standardisation of test methods and non-representative sampling, while it was acknowledged that representative sampling is often impossible or impractical [27].

Recently, a very high frequency of BZ-resistance alleles in *Haemonchus* spp. and *Trichostrongylus* spp. was detected in Styria, south-eastern Austria, indicating that this drug class was no longer efficient on any of the sampled farms [16]. The occurrence of moxidectin (MOX)-resistance was also suggested [28].

The aim of our study was to obtain updated information on the occurrence of AR in different trichostrongylid species that infect sheep in Austria by performing FECRT and larval cultures. To achieve this, two studies were performed in different federal states of Austria. In the first study, only two anthelmintic compounds (the BZ fenbendazole (FBZ) and/or the macrocyclic lactone MOX) were used. For study 1, we hypothesised that a high level of BZ-resistance is present and that MOX-resistance can be observed on Austrian farms. In the second study, a wider variety of compounds and products were applied so that all anthelmintic groups that were available for sheep in Austria were tested. For study 2, we hypothesised that routine treatments on Austrian farms are often not effective.

## Materials and methods

In the period from autumn 2018 to autumn 2020, 32 farms were examined and FECRTs for the detection of AR of various compounds were performed on 30 of these farms in two studies.

In study 1, FBZ and MOX were applied. FBZ was chosen to gather up to date information on the phenotype of BZ-resistance in Austria in order to complement recent findings that were only focused on the genotype of BZ-resistance [16]. Special attention was also paid to MOX, as it was proposed to still be efficacious when moderate resistance against other macrocyclic lactones (ML) is already present [22]. Thus, by focusing on the efficacy of this compound, we aimed to get a better impression of the overall progression of ML resistance in Austria.

In study 2, different factors required a change of study design: (1) the goal was to observe the efficacy of compounds applied in routine treatments. Therefore, we did not suggest a certain product. (2) Motivation of veterinarians and farmers to participate in our study was increased when they had a free choice to decide which anthelmintic compound was used. (3) Difficulties were encountered with the design of study one (initially planned as a randomised approach including control groups). Thus, the second study followed a more naturalistic (field-based) approach where the decision on compounds was not influenced by the investigators but only made by the attending veterinarian and the farmer. Besides BZ, ML (MOX, ivermectin (IVM), doramectin (DOR)) and the rather new compound monepantel (MON) were applied.

### Farms and animals

All of the farms examined had pasture access. A further prerequisite for participation was that no deworming was performed for at least three months before sampling. Animals over the age of 6 months were included. Sheep were kept for wool production, landscape conservation, breeding, and meat production. A combination of all of these purposes was often present on farms.

All farms from study 1 were organised in the same network for the breeding of Tyrolian mountain sheep, and selection was based on the interest of the farmers in participating, which was partly driven by experience of treatment failure on these farms.

In study 2, the majority of farms were consulted by the animal health service of Salzburg. An information mail about anthelmintic resistance was sent out by the animal health service encouraging farms to participate, and as many farms as possible were included. Selection was partly based on the practicability of visiting these farms and on the interest of the attending veterinarians. Additional farms not organised in the animal health service also participated, since they had observed treatment failures in their flocks. They were not visited, but samples were submitted by the attending veterinarians according to instructions. The main differences between the studies as well as further information on the study design are shown in Table 1.

**Table 1:** Farms and animals included in both studies as well as further details on study design.

	Study 1	Study 2
Time period of first examination	September–October 2018	Nov. – Dec. 2019 except #31 (Feb. 2020) and #32 + #33 (Sept. – Oct. 2020)
Min./max. number of animals examined/farm	10–70	10–40
Number farms/animals examined	13/500	19/375
Breed	Tyrolian mountain sheep	Various sheep breeds
Number farms/animals included in FECRT	11/126	19/263
Threshold EPG for inclusion in FECRT	≥100	≥50
Number of animals with EPG ≥ 200	93 (73%)	168 (63.9%)
Treatment decision by	Institute of Parasitology in consultation with attending veterinarian and farmer	Attending veterinarian and farmers
Treatment and sampling performed by	A project team member (FU)	The attending veterinarian, a project team member (VF, MHR or WK) or the farmer during the presence of a project team member (VF, MHR or WK).
Drug provided by	Institute of Parasitology, Vetmeduni Vienna	Attending veterinarian
Anthelmintic compounds used	MOX (Cydectin^®^, Elanco) 0.2 mg/kg BW and/or Fenbendazole (FBZ) (Panacur^®^ Suspension 2.5%, MSD) 5 mg/kg BW	Different compounds and formulations of the groups of BZs; MLs as well as MON (see Table 3)
Farms not visited but samples sent in. Sampling and treatment performed by attending veterinarian	–	*N* = 6 (#26, 27, 32, 31, 33)

### Faecal egg count reduction test

In study 1, faeces were collected rectally and individual samples were examined on the same day by Mini-FLOTAC [7], with a detection limit of 5 eggs per gram (EPG) of faeces, using a sodium chloride flotation solution (FS2, specific gravity = 1.200).

Based on the result of the egg counts, animals were allocated to treatment groups (Tables 1 and 2).

**Table 2:** Data about sheep farms included in study 1, anthelmintic drug applied, and number of animals included in the respective group (FBZ = fenbendazole; MOX = moxidectin); result of the FECRT. Classification: R = resistant; SR = suspected resistance; S = susceptible. Status in square brackets = number of animals in treatment group < 10. EZR = egg count reduction.

Farm #	Region/lowland or alpine pasture/frequency of deworming/contact with goats	Group/no. of animals included	Mean EPG value before/after treatment	EZR paired with individual efficacy (95% CI)	EZR paired (95% CI)	Status
1	Tyrol/alpine/2–4/yes	FBZ/12	620/62	95 (87–99)	90 (88–92)	SR
2	Tyrol/alpine/2–3/yes	MOX/7	605/11	98 (87–100)	98 (97–99)	[SR]
3	Tyrol/alpine/2–4/yes	MOX/12	385/114	79 (53–97)	70 (66–74)	R
4	Tyrol/alpine-fenced field/3–4/yes	MOX/12	1147/41	96 (87–99)	96 (96–97)	SR
5	Tyrol/alpine/2–3/no	FBZ/9	302/14	96 (92–98)	95 (93–97)	[S]
6	Tyrol/alpine/2–3/no	MOX/9	419/8	97 (87–100)	98 (97–99)	[SR]
7	Tyrol/alpine/2–3/no	MOX/10	306/3	99 (97–100)	99 (98–100)	S
9	Tyrol/alpine/2–3/yes	MOX/11	359/1	100 (99–100)	100 (99–100)	S
10	Styria/lowland/2–3/no	MOX/10	568/12	98 (92–100)	98 (97–99)	S
12	Styria/lowland/2–3/yes	FBZ/13	874/456	48 (23–66)	48 (44–51)	R
		MOX/13	1088/241	84 (61–96)	78 (76–80)	R
13	Tyrol/alpine/2–3/yes	MOX/8	308/2	99 (98–100)	99 (98–100)	[S]

Due to small flock sizes and/or low egg excretion levels, only eight groups included 10 or more animals each, while in four groups fewer animals were included (Table 2). Pregnant ewes were excluded from the FBZ groups. Faeces were examined on the day of sampling and animals were treated one day after faecal examination. Before treatment, the applicators were calibrated, and the animals were weighed on a portable scale (Soehnle Professional 2755, Soehnle Industrial Solutions GmbH, Backnang, Germany) to allow for body mass-based treatment. On farm 12, two compounds were applied and animals were allocated to the groups by random numbers. On day 14 after treatment, faecal samples were collected and individual egg counts were again obtained with Mini-FLOTAC with the same protocol as used before.

In study 2, no intervention in the treatment decision of the responsible veterinarian was made. However, treatment and sampling were supervised by a team member or an expert (Table 1). Attention was paid to the fact that no expired drugs were used. Animals were weighed to ensure that they received the correct dose of the drug, irrespective of the routine practices on the farms. This was either done with a portable scale or on scales provided on the farms. Only on farms 31 and 32 was the weight of animals estimated by the veterinarian and the dosage of the anthelmintic compound was adjusted to a slightly higher weight than estimated. The prescribed anthelmintic drug was applied to all animals (Tables 1 and 3) immediately after faecal sampling and body weight determination/estimation. The faeces of animals included in the FECRT (Tables 1 and 3) were examined within three days after sampling. Until examination, the samples were vacuum packed [24] and stored below room temperature to prevent egg development.

**Table 3:** Data about sheep farms included in study 2; anthelmintic drug applied and number of animals included in the respective group (FBZ = fenbendazole; ABZ = albendazole; IVM = ivermectin; DOR = doramectin; MOX = moxidectin; MON = monepantel) and result of the FECRT. Dosage applied: * ≈ 5 mgFBZ/kg BW; ** ≈ 5 mgABZ/kg BW; *** ≈ 3.8 mg ABZ/kg BW; + ≈ 0.2 mg IVM/kg BW; ++ ≈ 0.2 mg MOX/kg BW; ND = no data. Region: SZB: Salzburg, LA: Lower Austria. Classification: R = resistant; SR = suspected resistance; S = susceptible. Status in square brackets = number of animals in treatment group < 10. EZR = egg count reduction.

Farm #	Region/lowland or alpine pasture/frequency of deworming/contact with goats	Group/no. of animals included	Compound/dosage	Mean EPG value before/after treatment	EZR paired with individual efficacy (95% CI)	EZR paired (95% CI)	Status
14	SZB/lowland/2/yes	FBZ/5	*Panacur^®^ 250 mg tablets/0.5 tablet/25 kg*	1764/399	69 (35–98)	77 (75–80)	[R]
15	SZB/alpine/2/ND	FBZ/9	*Panacur^®^ 250 mg/bolus; 1 bolus/50 kg i.r.	1665/1856	57 (30–90)	0 (0–2)	[R]
16	SZB/alpine/2/ND	ABZ/13	**Albendazole 10% Suspension aniMedica/0.5 mL/10 kg	105/0	100 (99–100)	100 (99–100)	S
17	SZB/lowland/3–4/ND	ABZ/6	**Albendazole 10% Suspension aniMedica/0.5 mL/10 kg	514/255	52 (25–87)	50 (44–57)	[R]
18	SZB/lowland/2-3/yes	ABZ/16	***Valbazen^®^ 1.9%/1 mL/5 kg	1186/186	89 (83–94)	84 (83–86)	R
19	SZB/alpine/1–3/ND	ABZ/17	**Albendazole 10% Suspension aniMedica/0.5 mL/10 kg	579/372	61 (40–77)	36 (31–40)	R
		MOX/16	^++^Cydectin^®^/1 mL/5 kg p.o.	215/0	100 (100–100)	100 (100–100)	S
20	SZB/lowland/2/ND	IVM/13	^+^Noromectin^®^/0.5 mL/25 kg	888/455	53 (30–79)	49 (45–52)	R
21	SZB/alpine/depending on fecal examination/ND	MOX/11	^++^Cydectin^®^/1 mL/5 kg p.o.	424/67	86 (62–98)	84 (81–87)	R
22	SZB/lowland/2/ND	MOX/12	^++^Cydectin^®^/1 mL/5 kg p.o.*	1813/43	99 (92–100)	98 (97–98)	S
23	SZB/lowland/3–4/ND	MOX/19	^++^Cydectin^®^/1 mL/5 kg p.o.	471/3	100 (100–100)	99 (99–100)	S
24	SZB/lowland/2/ND	MOX/11	^++^Cydectin^®^/1 mL/5 kg p.o.	326/6	100 (97–100)	98 (97–99)	S
25	SZB/alpine/1-3/ND	MOX/13	^++^Cydectin^®^/1 mL/5 kg p.o.	1897/4	100 (100–100)	100 (100–100)	S
26	LA/lowland/ND/ND	MOX/15	^++^Cydectin^®^/1 mL/5 kg p.o.	1241/1	100 (100–100)	100 (100–100)	S
27	LA/lowland/ND/ND	ABZ/10	**Albendazol 10% Suspension aniMedica/0.5 mL/10 kg	1419/70	97 (93–99)	95 (94–96)	S
		MOX/7	^++^Cydectin^®^/1 mL/5 kg p.o.	296/0	100 (99–100)	100 (99–100)	[S]
28	SZB/lowland/1/ND	MON/15	^+++^Zolvix^®^/2.5 mg/kg p.o.	728/0	100 (100–100)	100 (100–100)	S
29	SZB/lowland/2/yes	MON/11	^+++^Zolvix^®^/2.5 mg/kg p.o.	818/383	87 (59–99)	53 (49–57)	R
31	SZB/ND/ND	ABZ/18	**Albendazole 10% Suspension aniMedica/0.5 mL/10 kg	440/124	79 (63–91)	72 (69–75)	R
32	LA/lowland/2/ND	MOX/12	^++^Cydectin^®^/1 ml/5 kg p.o.	1820/428	89 (66–99)	77 (75–78)	R
33	Styria/lowland/2/yes	DOR/14	Dectomax^®^/0.2 mg/kg i.m.	1190/140	92 (77–99)	88 (87–89)	R

### Larval culture and larval differentiation

Before treatment, all samples positive for strongyle eggs from one farm were pooled for larval culture. After treatment, the positive samples of each farm were pooled per treatment group. Faeces were mixed with water and vermiculite and incubated at 25 °C for 13 days. On day 14, the third-stage larvae were harvested and identified (≈100 larvae per coproculture) using the identification key developed by van Wyk et al. [36].

### Statistical analysis

For calculation of the FECR and corresponding credibility intervals, the web interface (https://www.math.uzh.ch/as/index.php?id=software_as00) based on the R package eggCounts 2.3 was used [31, 39]. “eggCounts” uses a hierarchical Bayesian model to cover the different levels of variation in egg count data. These include: (i) a binomial distribution of EPGs to cover differences between true EPG and observed EPG due to dilution and counting before and after treatment; (ii) a Poisson model to model true EPGs, which covers random distribution of eggs in the faecal sample; (iii) a gamma distribution to model overdispersed egg shading intensity between animals both before and after treatment; (iv) individual treatment efficacies for each animal based on a random effect model with treatment efficacies following another gamma distribution. Calculations were carried out using pre- and post-treatment egg counts with the standard “two sampled paired” (without allowing individual treatment efficacies) and “two samples paired with individual efficacy” parameters. No zero-inflated distributions were considered since only positive animals were included in the FECRT. The final interpretation of efficacy was based on “two samples paired with individual efficacy”. eggCounts uses a Bayesian approach and Markov chain Monte Carlo sampling to estimate model parameters from the data. The estimate for the FECR is the mode of the posterior FECR distribution, the 95% credibility limits from the 2.5% and 97.5% quantiles of the distribution [39].

Definition of AR was based on Coles et al. [4], where resistance is considered to be present when egg count reduction is less than 95% and the lower CL is less than 90%, and suspected resistance (SR) is present when only one of the two criteria is observed. The difference between the two calculation standards of eggCounts was further analysed (Supplementary Files).

Testing for significance between groups was carried out by applying the Mid-P exact test provided by OpenEpi (https://www.openepi.com). The difference was considered significant at *p* < 0.05.

## Results

### Faecal egg count reduction test

In study 1, BZ resistance, suspected resistance and susceptibility were detected on one farm each. However, on the farm with susceptibility, only nine animals were examined (Table 2). MOX resistance was detected on two, suspected resistance on three, and susceptibility on four farms. Amongst the groups with susceptibility and suspected resistance, three consisted of <10 animals (Table 2).

In study 2, BZ resistance was observed on six farms (of which in three <10 animals were examined) and susceptibility on two farms. MOX resistance was observed on two farms and susceptibility on six farms, of which in one <10 animals were examined. On both farms where IVM or DOR were tested, resistance was present (Table 3). Monepantel resistance and susceptibility were detected on one farm each (Table 3).

### Larval differentiation

**Figure 1:**
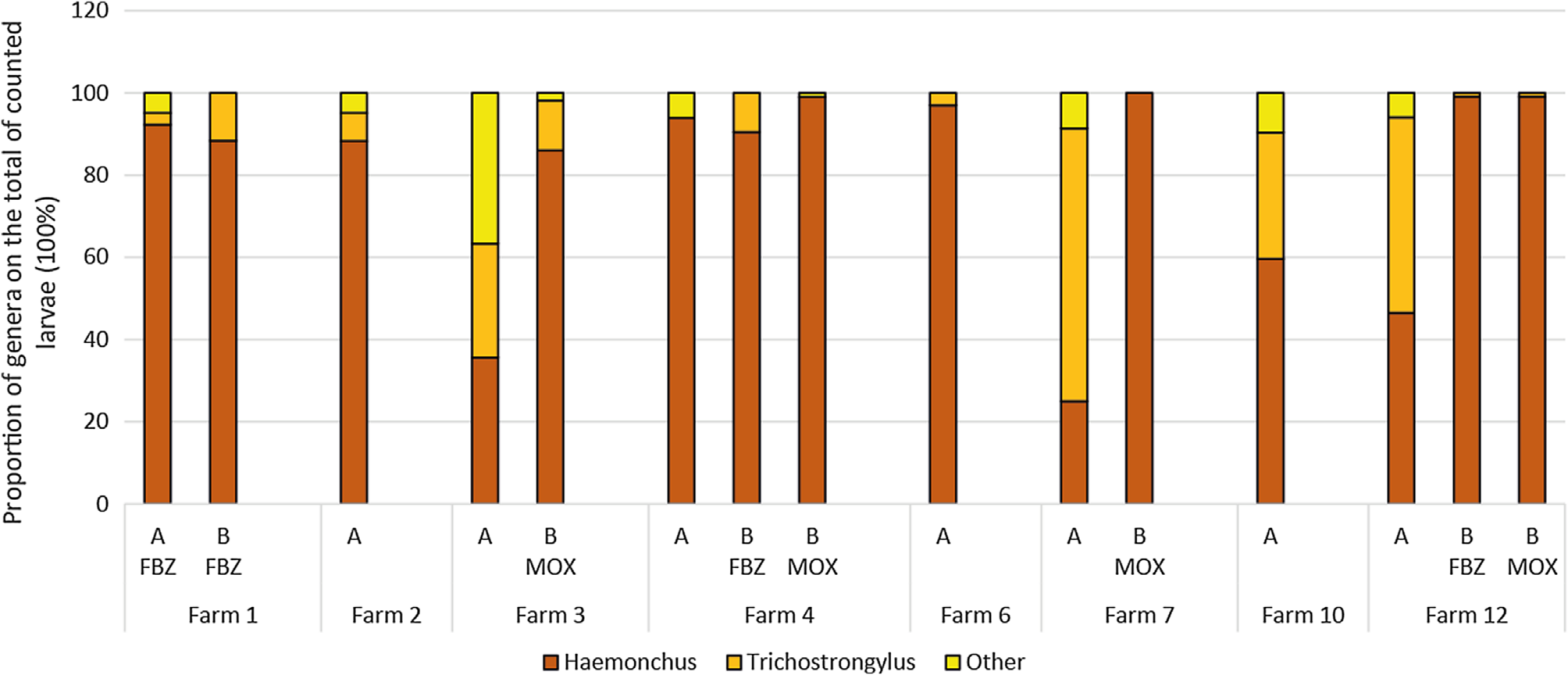
Results of larval differentiation in study 1 shown as proportions of different genera. Each column represents 100% of counted larvae. A = before treatment (pooled samples of whole farm), B = after treatment (pooled samples of the respective treatment group). Other = *Teladorsagia, Chabertia, Oesophagostomum*.

For unidentified reasons, the larval cultures did not yield sufficient larvae in all cases. In study 1, for eight farms, sufficient (*n* = 100) larvae could be harvested for larval differentiation before treatment. On five of these, *Haemonchus* spp. larvae were predominant, and on three *Trichostrongylus* larvae were determined in the same or larger numbers (Fig. 1). Other genera (*Chabertia*, *Oesophagostomum*, *Cooperia*, *Teladorsagia*) occurred very rarely and only at low frequency. From five farms and in seven groups, more than 100 larvae post treatment were harvested. Post treatment, the relative frequency of *Haemonchus* spp. increased in all MOX treatment groups (significantly on farms #3, #7 and #12; *p* < 0.001) and in the FBZ treatment group of farm #12 (*p* < 0.001).

**Figure 2:**
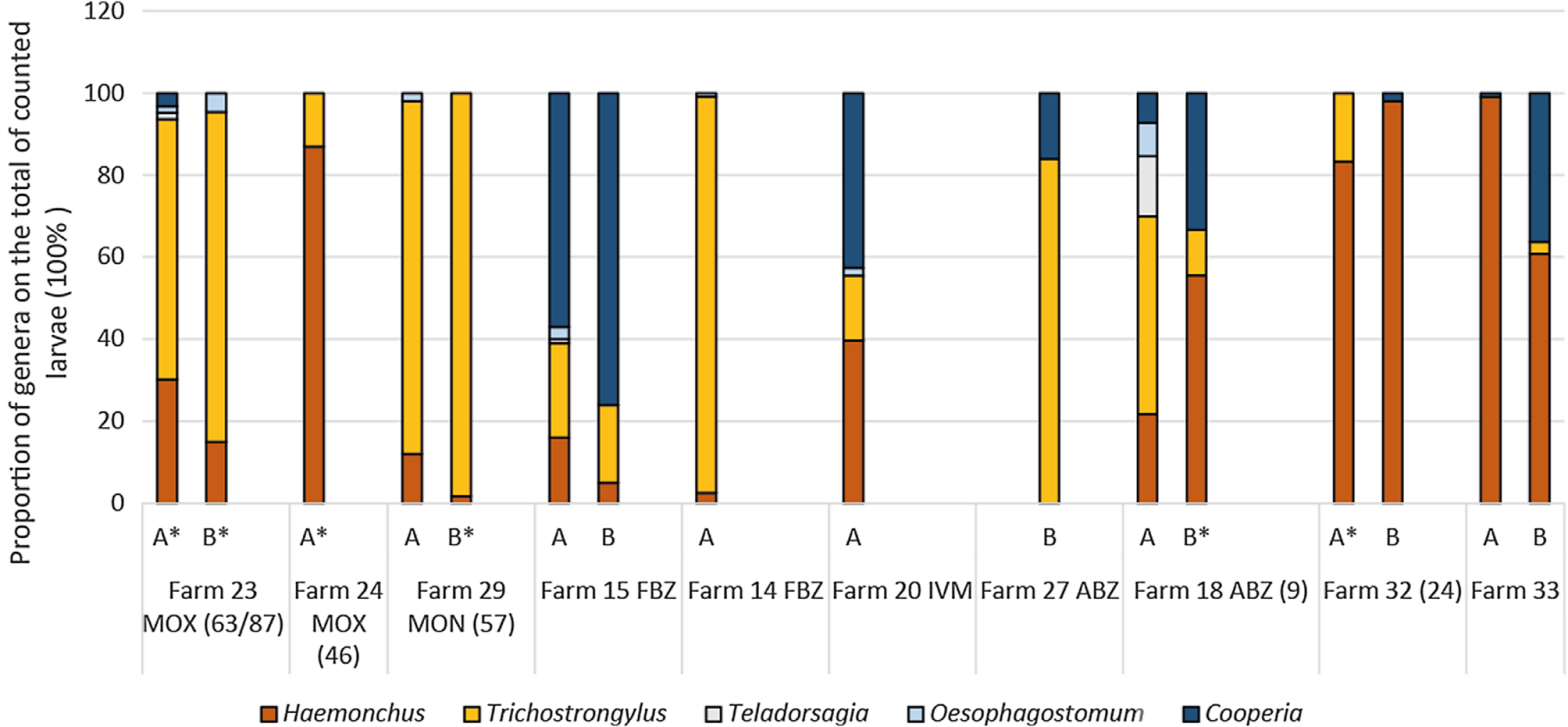
Results of larval differentiation in study 2 shown as proportion of different genera. Each column represents 100% of counted larvae. A = before treatment (pooled samples of whole farm), B = after treatment (pooled samples of the respective treatment group). If less than 100 larvae were differentiated, this is marked by an asterisk, and number of larvae that were differentiated is given in brackets.

In study 2, larval cultures only yielded sufficient larvae for seven farms before treatment and three farms post treatment (Fig. 2). Before treatment, on two farms *Trichostrongylus* was predominant, on two *Haemonchus*, on one farm *Cooperia,* and on farm #18 four genera with no clear dominance were counted. Post treatment on farm #15 the predominance of *Cooperia* significantly increased from 57% to 76% after treatment with FBZ. On farm #33, where almost only *Haemonchus* (99%) was detected before treatment, 36% *Cooperia* were counted after treatment (this difference was significant; *p* < 0.001). On farm #27 after treatment with BZ, *Trichostrongylus* was predominant with 84% (no pre-treatment data were available). Additional results from cultures with less than 100 larvae counted are shown in Figure 2.

## Discussion

### Anthelmintic resistance on the farms examined

Reduced efficacy of all anthelmintics available for sheep in Austria was observed in our studies. We were able to provide the first description of MON resistance and the first clear evidence of MOX resistance in Austria. MOX resistance was previously suspected for one farm [28] but could not be confirmed so far.

It cannot be estimated how fast these resistances have developed in Austrian nematode populations and what the actual prevalence of AR is, since there is a lack of previously generated representative data on AR in Austria and these were also not provided within this study. There have hardly been any systematic studies performed which would allow to draw such conclusions in other European countries either [27]. However, for reliable estimates a longer-term approach of this kind would be necessary. This can be seen in Norway, where a representative sampling yielded results that differed substantially from data generated by convenience sampling [11]. In addition, in both of our studies, convenience samples were taken. Thus, the non-representative sampling with voluntary participation probably resulted in a selection bias with an overrepresentation of farms that had already experienced treatment failure, or farms that were especially interested in sustainable parasite control and thus have management strategies that are not representative for Austria. Furthermore, the aims and study design of both studies presented here differed so that the degree of comparability of the two studies is low. For example, only two compounds provided by us were tested in study 1, while in study 2, six different compounds and seven products provided by the attending veterinarian were used. Nonetheless, the procedure of the FECRT itself was very similar (e.g. application of the drug through or under supervision of a team member or expert after the weighing of animals, the use of Mini-FLOTAC on individual samples and the use of two standards of eggCounts for the calculation of FECR). Thus, although the non-representative sampling and different study design does not allow a comparison of the two studies, for simplification we combine observations of both studies to form a general picture of AR in Austria in the following discussion.

Benzimidazole resistance in Austria was already described in 1996 [15], and this matches the observation of increasing BZ resistance worldwide for almost four decades [6]. It also matches our previous observation of high levels of BZ-resistance alleles in Austria [16]. These observations notwithstanding, susceptibility to BZ was observed on three farms. The frequency of BZ resistance of 64% in our study is comparable to the observations of most Central and some Southern European studies, while especially Italy and Spain showed a considerably lower prevalence. Northern and Eastern European countries also tend to have lower frequencies of BZ resistance [27].

We only tested one sheep farm each for efficacy of IVM and DOR, and in both cases resistance was detected. However, this does not allow us to draw generalised conclusions about the efficacy of these drugs on Austrian sheep farms nor to compare it with European studies where up to 50% of farms were observed to show ML resistance [27]. It is currently assumed that MOX shows a certain degree of cross-resistance with other MLs, but that it might still be efficacious on some farms where ML resistance has already appeared [22]. Although resistance to MOX might develop rather slowly [22], it appears to already be quite progressed in Austria, since the drug was introduced in 1998, as 24% of the tested farms showed a resistant nematode phenotype. Only a few European studies have investigated MOX on a larger number of farms. The frequency of MOX resistance was slightly higher in the UK and the Netherlands (37% and 34%) and comparable in Germany (19%), while no MOX resistance was described in Italy [27]. In contrast to MOX, MON resistance seems to develop rather rapidly [32]. In line with this (and despite the fact that MON was introduced in Austria only in 2010), we already observed MON resistance in one of the two tested sheep farms. This seems highly alarming as AR against all registered compounds available in Austria was thus observed (levamisole is not on the market at present). MON resistance in European countries has also already been observed in Belgium, the Netherlands, Switzerland, and the UK [27].

To conclude, all of the hypotheses made for study 1 and 2 were supported by our findings: high frequencies of BZ resistance, the presence of MOX resistance and ineffective routine treatments in the field. The latter might be partly based on the fact that farmers preferred to use cheaper compounds, with BZs frequently applied. Obviously, veterinarians and farmers were not aware of the high frequency of AR in BZ drugs. However, AR also occurred on farms where the more expensive compounds MOX and MON were used. We did not document management factors in detail, but as whole flock treatments and dose-and-move strategies were common practice on the investigated farms, probably most veterinarians and farmers had not been informed about sustainable control methods. Consequently, methods were applied that led to a strong selection for AR. Thus, it will be necessary to develop effective communication channels so that sustainable control strategies will be readily adopted by farmers and veterinarians [35, 37]

### Composition of trichostrongylid species as detected by larval culture

*Haemonchus* and *Trichostrongylus* were the most frequently detected genera in this study. While *Haemonchus* spp. were amongst the most frequently counted larvae in study 1 (Styria and Tyrol), on five farms in study 2 (mostly in Salzburg), *Trichostrongylus* larvae predominated. It is important to note that the eggs shed by a species do not directly correlate with the number of individuals in the host. *Haemonchus* spp. in particular are more fecund than most other trichostrongylid species [14]. Another factor involved in fecundity is the host itself, since immunity, health and genetics can strongly influence the number of eggs shed by intestinal worms [26]. In addition, samples in which *Trichostrongylus* dominated were collected in November/December, whereas those samples in which *Haemonchus* was present were mostly collected in September and October. The mean temperatures in Austria in September and October 2018 were 14.2 °C and 9.9 °C, respectively, while they dropped to 3.9 °C and 0.7 °C in November and December 2019 (Central Institute for Meteorology and Geodynamics (ZAMG); https://www.zamg.ac.at). Thus, the temperature-sensitive *Haemonchus* had probably already started to enter hypobiosis at the later sampling. It therefore cannot be concluded that *Haemonchus* occurs more often in Styria and Tyrol and less often in Salzburg. Additionally, this as well as previous studies in Austria did not generate geographically representative data, as convenience samples were examined. Similar reasons prevent us from concluding with certainty that the prevalence of *Haemonchus* and *Trichostrongylus* in Austria has increased in recent years. In general, *Haemonchus* was considered to be of no relevance for alpine regions in the past [12]. This is clearly not the case anymore, as *Haemonchus* occurred on all examined farms in the present study, independently of the pasturing type. The spatial distribution of *Haemonchus* is dependent on many factors, amongst them temperature, with a clear preference for a warm climate [21]. Climate change might lead to a shift in the composition of pasture-borne worm populations in livestock and might have led to a higher prevalence of *H. contortus* in regions where this species was previously underrepresented [33]. In 1977, *Haemonchus contortus* was hardly detected in sheep from Austria [13]. Since 1880, average temperatures in Austria have increased by about 2 °C [1]. Indeed, the mean temperature in Austria throughout the 1970s was 1.73 °C lower than throughout the 2010s (6.17 °C vs. 7.9 °C (ZAMG https://www.zamg.ac.at). However, we assume that the development of AR is also a strong factor for the unexpectedly high prevalence of *Haemonchus* (and *Trichostrongylus*) in our study, as they were clearly the genera that dominated post treatment. Through its high fecundity and high genetic diversity, *Haemonchus* has the ability to develop AR particularly quickly [18]. Interestingly, in a recent study, we found few BZ-resistance alleles in *Teladorsagia* from Austria, while they occurred in high frequencies in *Haemonchus* and *Trichostrongylus* [16]. These observations match the results of a low *Teladorsagia* prevalence in the present study.

### Faecal egg count reduction test

The FECRT has the advantage of being a universally applicable test for detecting AR, but has the disadvantage of low sensitivity. Its comparability is limited by the fact that there are several ways to perform the test and interpret the results [17], although guidelines exist [5]. The present study deviated from the WAAVP guidelines, mainly due to the fact that farm sizes were too small and not enough animals reached a sufficient EPG, and consequently not all treatment groups had at least 10 animals. Also, one third of individuals included did not meet the WAAVP-requirements of an EPG of 200 [5]. However, we performed the Mini-FLOTAC technique with a detection limit of 5 EPG and thus achieved higher analytic sensitivity and accuracy than the McMaster technique, which was recommended in the WAAVP-guideline [4]. Tests with higher accuracy might make it possible to obtain valid data even when animal numbers and/or egg counts are low [19]. It has been indicated that new WAAVP guidelines will even accept a raw egg count of 200 per treatment group [17], and this requirement was met in all of our groups. On farms with low animal numbers, instead of performing FECRT, molecular tests like resistance allele-analysis or laboratory tests like the egg hatch test might be better alternatives for detecting AR against BZ. However, for ML or MON, technically more complicated tests such as the larval development test are required. Clustering small farms that are from the same region and that are grazing the same pasture for a FECRT (e.g. in transhumance systems) might be another possibility to deal with the problem of low animal numbers on individual farms. The use of composite and repeated samplings for small farms should also be considered. Including a control group in the FECRT is the current recommendation of the WAAVP guidelines. Although we tried to include control groups in study 1, they ultimately could not be used for FEC calculation. Interestingly, some authors even observed that omitting the control group renders more reliable results [10]. Various methods have been proposed to calculate the percentage of egg reduction on farms. We used two standards of eggCounts for the calculation of FECR. They differed clearly when variability within a farm was high (Supplementary Files) and as the standard with individual efficacy is considered to give a more precise estimate of FECR, this might be the preferred standard to apply [39]. As protocols and methods for the detection of AR vary considerably and are hardly standardised, Working Group 1 of the COST action COMBAR (https://www.combar-ca.eu/) recently harmonised the current protocols for the diagnosis of AR, which will allow for better comparability of test results (https://www.combar-ca.eu/sites/default/files/FECRT_PROTOCOL_sheep_goats_March%202021.pdf).

## Conclusion

Multispecific resistance of trichostrongylids has been detected on Austrian sheep farms. The most abundant and resistant genera were *Haemonchus* and *Trichostrongylus*. Best practice advice that can be easily implemented should thus be communicated to practitioners and farmers as soon as possible. We also wish to point out that monitoring drug efficacy on small farms (<40 animals) is important for obtaining an informative picture of AR in countries where most farms are small. Guidelines that consider different farm sizes would help to comprehensively monitor anthelmintic efficacy worldwide.

## Supplementary Material

Supplementary material is available at https://www.parasite-journal.org/10.1051/parasite/2021048/olm*Supplementary Data*. Comparison of different standards of eggCounts 2.3.**Supplementary Figure 1:**
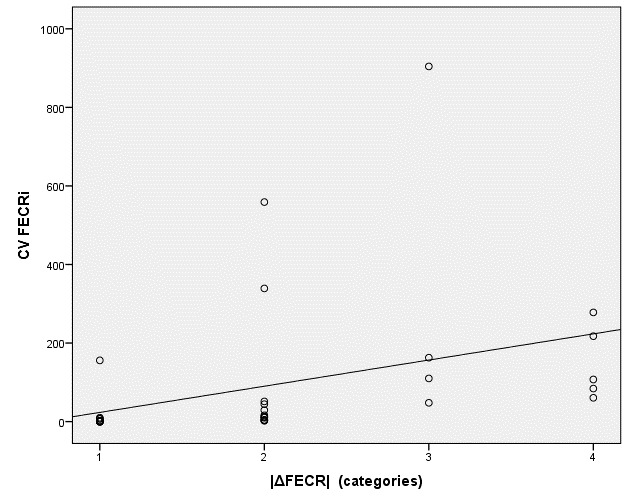
Scatter plot of the coefficient of variation of individual egg count reduction on farms (CV FECRi) plotted against the difference between the two calculation standards of eggCounts (|ΔFECR|-categories). Categorisation: 1 = Δ0; 2 = Δ1–5; 3 = Δ6–10; 4 = Δ > 10.

## Conflict of interest

The authors have no conflicts of interest to declare. MHR was employed at the animal health service Salzburg.

## Ethics approval and consent to participate

This investigation was discussed and approved by the institutional ethics and animal welfare committee of the Vetmeduni, Vienna in accordance with good scientific practice guidelines and national legislation (ETK-07/08/2018; study 1, ETK-185/11/2019; study 2). Written consent was obtained from all animal owners.

The number of farms with AR detected in study 1 was already included in a recent meta-analysis [27].
