# Supplementary material for: Multispecific resistance of sheep trichostrongylids in Austria
Source: Parasite. 2021 Jun 11;28:50. doi: 10.1051/parasite/2021048 (PMC8194391; doi:10.1051/parasite/2021048)
Supplement: Supplementary file 1 — Supplementary Data. Comparison of different standards of eggCounts 2.3. [file parasite-28-50-s1.pdf]

Comparision of different standards of eggCounts 2.3

1. **Methods**

The difference between the percentages of egg reduction between the two calculation methods (the standard "two sampled paired" and "two samples paired with individual efficacy") was expressed as the absolute number of the difference in the estimated FECR "|ΔFECR|". Also, for each animal the egg count reduction of individual animals (FECRi) was calculated with the formula 100*(1-egg count after treatment /egg count before treatment). For each farm, the coefficient of variation of FECRi was calculated (CV FECRi). Then, |ΔFECR| on each farm was plotted against the CV FECRi for the same farm. The Spearman correlation coefficient between "CV FECRi" and |ΔFECR| was computed (two-tailed, pairwise exclusion of missing data) with SPSS Statistics 24 (IBM GmbH, Ehningen, Germany). Furthermore, a scatter plot of both variables was generated.

2. **Results:**

In most of the calculations "FEC paired" yielded results similar to "FEC paired with individual efficacy". However, "FEC paired with individual efficacy" often resulted in higher efficacies when egg reduction in flocks was unevenly distributed. The |ΔFECR| on farms correlated positively with CV FECRi "Δ egg count standards" (Spearmann-Rho= 0.78; p= 0.000) (Supplementary Figure 1).

3. **Discussion**

The two standards of eggCounts for the calculation of FECR differed clearly when variability within a farm was high. As the standard with individual efficacy is considered to give a more precise estimate of FECR this might be the preferred standard to apply.
